# Comparative Analysis of Physiological and Biochemical Responses Between Compatible and Incompatible Graft Combinations of *Cyclocarya paliurus*

**DOI:** 10.3390/plants15101536

**Published:** 2026-05-18

**Authors:** Yiran Song, Xin Ma, Run Xu, Caowen Sun

**Affiliations:** 1 State Key Laboratory of Tree Genetics and Breeding, Nanjing Forestry University, Nanjing 210037, China; 18936965664@163.com (Y.S.); 18397022881@163.com (X.M.); xu18756246282@163.com (R.X.); 2 Co-Innovation Centre for Sustainable Forestry in Southern China, Nanjing Forestry University, Nanjing 210037, China; 3 National Key Laboratory for the Development and Utilization of Forest Food Resources, Nanjing Forestry University, Nanjing 210037, China

**Keywords:** *Cyclocarya paliurus*, graft compatibility, scion, enzyme activity, phenylpropanoids

## Abstract

*Cyclocarya paliurus* (Batal.) Iljinsk is a multipurpose tree species with great potential for development. To identify indicators of grafting compatibility during the healing process, two cloned genotypes, CR4 and CR5, were used as scions, grafted onto seedling rootstocks derived from the Guangxi provenance. Following branch grafting, samples from the graft union were collected at 0, 20, 40, and 60 days. Physiological–biochemical indicators, including soluble sugars, soluble proteins, starch, peroxidase (POD) activity, polyphenol oxidase (PPO) activity, phenylalanine ammonia-lyase (PAL) activity, as well as flavonoid and phenylpropanoid contents, were analyzed. The graft survival rate of the CR4 combination (60%) was significantly higher than that of CR5 (35%). Significant differences in key physiological indicators were observed between the two scion–rootstock combinations. Analysis revealed that soluble sugar and soluble protein levels, along with POD and PAL activities in the scion during the early grafting stage, significantly influenced the final survival rate. Furthermore, lignans, lignin precursors, and several flavonoid compounds were found to accumulate preferentially at the graft union of the CR4 combination, which exhibited higher compatibility. These findings provide a physiological and biochemical foundation for selecting compatible scions and advancing clonal cultivation of *C. paliurus*.

## 1. Introduction

*Cyclocarya paliurus* (Batal.) Iljinsk, a deciduous tree species of the genus *Cyclocarya* within the Juglandaceae family, is indigenous to southern China. This multipurpose tree is valued for its medicinal properties, timber utility, and ornamental appeal [1,2]. It exhibits rapid growth and produces straight trunks suitable for furniture-making, while its distinctive coin-like fruits contribute to its ornamental potential. Additionally, the leaves of *C. paliurus* hold significant pharmaceutical value, with demonstrated therapeutic potential in managing chronic conditions such as diabetes [3,4] and hyperlipidemia [5,6]. However, limited progress has been made in the propagation techniques, such as grafting and tissue culture in *C. paliurus,* which hinder the propagation of superior varieties.

Previous research [7] reported that *C. paliurus* grafting success was substantially higher on conspecific rootstocks than on heterograft combinations, while grafting survival rate on conspecific rootstocks was still unstable (50–80%). There were highly significant differences in survival rates from different *C. paliurus* scion–rootstock combinations [8]. Our prior research identified leaf total flavonoid content as a potential biochemical marker for assessing graft compatibility in this species [9]. And phenylpropanoids were also found to accumulate in compatible graft unions [8]. Following grafting, a cascade of physiological and metabolic alterations takes place at the union, among which certain key metabolites may exert a decisive influence on the final grafting survival rate [10,11]. The compatibility between scion and rootstock can be reflected, to some extent, by physiological changes in nutritive reserves and defense-related enzyme activities at the graft union [10,12].

Despite the fact that *C. paliurus* scion–rootstock combinations significantly influenced graft survival rate [4,5,6], previous research only identified differentially expressed metabolites after graft healing [5]. During the healing process, physiological, biochemical, and phenylpropanoid variations between *C. paliurus* graft combinations of differing compatibility remain unclear. The mechanism of *C. paliurus* graft incompatibility urgently needs to be revealed. Our aim is to uncover the physiological, biochemical, and phenylpropanoid variation between compatible and incompatible *C. paliurus* graft combinations during the graft healing stage, and to reveal the potential mechanism leading to graft incompatibility of *C. paliurus*.

Two *C. paliurus* clones with contrasting grafting survival rates were selected as scions. Both were grafted onto the same rootstock provenance, establishing two distinct graft combinations. The physiological and biochemical dynamics at the graft union were systematically compared throughout the healing process. This comparative approach aimed to elucidate the relationship between key physiological–biochemical characteristics and graft compatibility. The findings are expected to establish a foundation for optimizing scion selection in the graft-based cultivation of *C. paliurus*.

## 2. Materials and Methods

### 2.1. Plant Materials and Experimental Site

The study was conducted at the Baima Teaching and Research Base of Nanjing Forestry University, located in Baima Town, Lishui District, Nanjing City, Jiangsu Province (119°09′ E, 31°35′ N). This area lies within the central subtropical zone. The local climate is characterized by an annual average temperature of 15.5 °C, a frost-free period of 237 days, an average annual sunshine duration of 2240 h, and an average annual precipitation of 1087.4 mm.

Two *C. paliurus* clones, CR4 and CR5, were used as scions. One-year-old seedlings derived from the Guangxi Zhuang Autonomous Region served as the uniform rootstock. In March 2024, branch grafting was performed to establish the two graft combinations (details provided in Table 1). For each combination, 40 grafted plants were prepared. Samples were collected from the graft unions at 0, 20, 40, and 60 days after grafting (Figure S1). At each time point, graft union tissues (including scions and rootstocks sampled at 5 cm from the graft union) were harvested from three randomly selected grafted seedlings of each graft combination. Immediately after collection, samples were flash-frozen in dry ice, finely ground in liquid nitrogen, and subsequently stored at −80 °C until further analysis.

### 2.2. Physiological and Biochemical Analyses

Soluble protein content: Fresh tissue (0.2 g) was homogenized in an appropriate extraction buffer and centrifuged at 9000 rpm for 5 min. The supernatant (0.2 mL) was mixed with 5 mL of Coomassie Brilliant Blue G-250 reagent (Macklin, Shanghai, China). Absorbance was measured at 595 nm using a spectrophotometer (Unicosh, Shanghai, China). Bovine serum albumin (Macklin, Shanghai, China) was used as the standard for generating a calibration curve, and the protein concentration was calculated accordingly.

Soluble sugar and starch content: Dried and powdered tissue (0.1 g) was extracted with 15 mL of 80% (*v*/*v*) ethanol in a boiling water bath for 30 min. After centrifugation at 3000 rpm for 5 min, the supernatant was collected and brought to a final volume of 50 mL. An aliquot (0.5 mL) of the extract was mixed with 5 mL of anthrone-sulfuric acid reagent. Following incubation, absorbance was measured at 625 nm. Glucose was used as the standard for quantifying soluble sugars. For starch determination, the insoluble residue after sugar extraction was hydrolyzed to glucose using sulfuric acid, followed by quantification with the same anthrone method.

Enzyme activity assays: The activities of peroxidase (POD), polyphenol oxidase (PPO), and phenylalanine ammonia-lyase (PAL) were determined using commercially available enzyme-linked immunosorbent assay (ELISA) kits (Nanjing Jiancheng Bioengineering Institute, Nanjing, China), according to the manufacturer’s instructions.

### 2.3. Phenylpropanoid Profiling

Phenylpropanoid metabolites were analyzed using high-performance liquid chromatography (HPLC; Agilent 1290 Infinity, Beijing, China) at Shanghai Applied Protein Technology Co., Ltd. [2]. Separation was performed on a C18 reversed-phase column (2.1 mm × 100 mm, 1.7 µm particle size; Waters, Ireland) with a mobile phase consisting of solvent A (25 mmol/L ammonium acetate and 25 mmol/L ammonium hydroxide in water) and solvent B (acetonitrile). A gradient elution program was employed as described by Sun et al. (2025) [8]. Data processing was conducted using the Gene Denovo platform (Guangzhou, China). Mass spectra were acquired in the range of 60–1000 *m*/*z* with a scan rate of 0.20 s/spectrum. Metabolites were identified by matching experimental spectra against the METLIN (http://metlin.scripps.edu) and MoNA (https://mona.fiehnlab.ucdavis.edu/) databases. Differential metabolites between groups were screened using a variable importance in projection (VIP) score ≥ 1 from an orthogonal partial least squares–discriminant analysis (OPLS-DA) model and a Student’s *t*-test threshold of *p* < 0.05. Cross-validation and permutation testing were carried out to test the results. False Discovery Rate (FDR) values were also used in metabolite analysis to decrease the risk of false positives. Identified metabolites were subsequently normalized for comparative analysis. The contents of flavonoids and phenylpropanoids were quantified by ion abundance.

### 2.4. Statistical Analysis

All data are presented as the mean ± standard deviation (SD). Statistical analyses were performed using SPSS software (version 22.0). Two-way analysis of variance (ANOVA) followed by Duncan’s multiple range test was used to determine significant differences among treatment means at a significance level of *p* < 0.05. Non-parametric tests are used when variances are heterogeneous.

## 3. Results and Analysis

### 3.1. Graft Survival Rate

**Figure 1:**
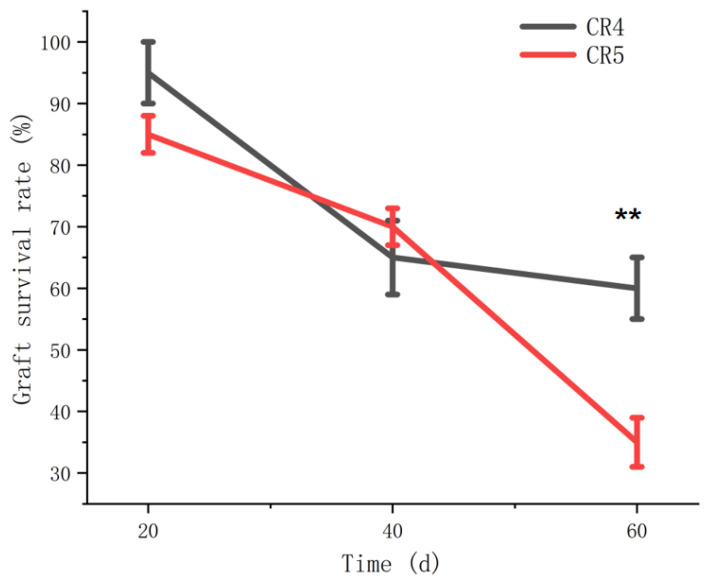
The survival rate of *Cyclocarya paliurus* (Batal.) Iljinsk graft combinations. ** indicates a highly significant difference at the *p* < 0.01 level at the same time point.

The final graft survival rate serves as a direct indicator of compatibility between the scion and rootstock. Survival rates for the two graft combinations (CR4 and CR5) were assessed at 20, 40, and 60 days after grafting. As shown in Figure 1, the survival rate for the CR4 combination was 95%, 65%, and 60% at 20, 40, and 60 days, respectively. In contrast, the survival rates for the CR5 combination were 85%, 70%, and 35% at the corresponding time points (Figure 1). Statistical analysis revealed that the overall survival rates between the two combinations differed significantly by 60 days. Notably, despite the survival rate for the CR5 combination (70%) being comparable to that of CR4 (65%) at 40 days, it declined sharply to 35% by 60 days. This final value was significantly lower than the 60% survival rate maintained by the CR4 combination.

### 3.2. Variance Analysis of Physiological–Biochemical Indicators Between Graft Combinations

The concentrations of key nutritional reserves (soluble sugars, starch, and soluble proteins) and the activities of major enzymes (PPO, POD, and PAL) were measured in the scions, graft unions, and rootstocks at 0, 20, 40, and 60 days after grafting for both combinations. The coefficient of variation (CV) for each indicator across time points is summarized in Table 1. Soluble sugar content exhibited the highest variability (CV = 0.34) at 40 days (CR4) and the lowest (CV = 0.09) at 20 days (CR5). Starch content showed the greatest variation (CV = 0.37) at 40 days (CR5) and the least (CV = 0.14) at 0 days of CR5. Soluble protein content varied most (CV = 0.32) at 40 days of CR5 and least (CV = 0.10) at 20 days of CR4. And soluble sugar content ranged from a maximum of 37.66 mg/g at 0 days to a minimum of 8.80 mg/g at 40 days. Starch content peaked at 28.13 mg/g (0 days) and reached its lowest level of 6.05 mg/g at 40 days. Soluble protein content increased over time, with a minimum of 25.63 mg/g at 40 days and a maximum of 85.30 mg/g at 60 days.

The activities of PPO, POD, and PAL also displayed significant temporal dynamics over the 60-day period. PPO activity showed the highest relative variation (CV = 0.37) at 20 days of CR5, and exhibited the lowest relative variation (CV = 0.07) at the same time point of CR4. While POD activity showed the highest relative variation (CV = 0.11) at 0 days of CR4, and exhibited the lowest relative variation (CV = 0.02) at 40 days of CR5. PAL activity CV was highest at 0.28 (20 days of CR4) and lowest at 0.07 U/g (20 days of CR5).

Two-way analysis of variance (ANOVA) was conducted to evaluate the effects of the primary factors and their interactions on the measured physiological–biochemical indicators, as detailed in Table 2. Two models were constructed. First, the effects of days after grafting (days: 0, 20, 40, and 60 days) and sampling site (scion and rootstock) were analyzed separately for the CR4 and CR5 graft combinations. Second, the effects of days and clones (CR4 and CR5) were analyzed for each of the two sampling sites. The results of ANOVA analysis indicated that days and clones significantly influenced PPO, POD, PAL, soluble sugar and starch of *C. paliurus* graft scions. And to graft rootstock, days and clones significantly influenced PPO, POD, PAL and soluble sugar at *p* < 0.01 level. On the other hand, PPO, POD, PAL, soluble sugar and starch of the CR4 graft combination were significantly regulated by days and site. PPO and POD of the CR5 graft combination were also significantly influenced by days and site. In addition, some variables require further nonparametric tests due to heterogeneity of variance.

### 3.3. Dynamics of Nutrient Indicators in Graft Combinations

**Figure 2:**
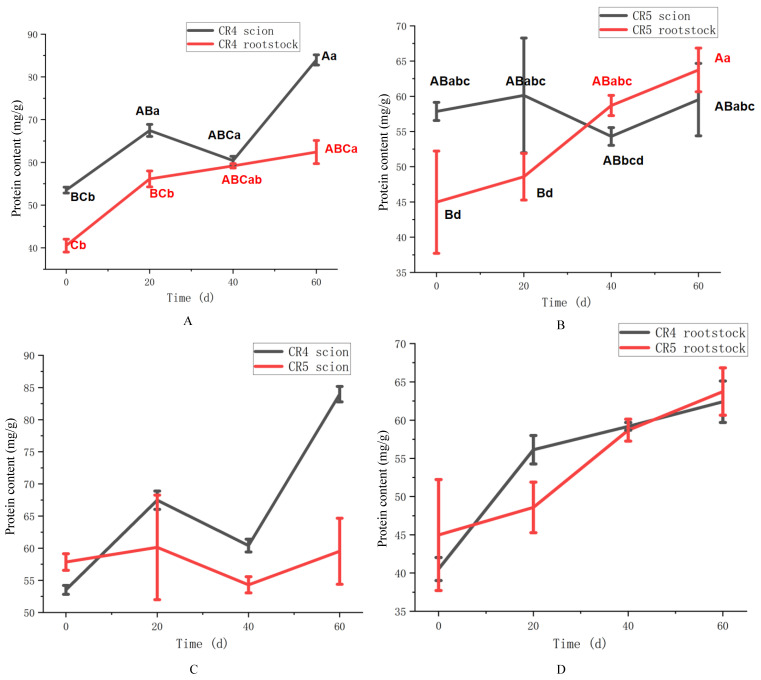
Soluble protein content of CR4 and CR5 graft combinations. (**A**) Soluble protein content of the CR4 combination. (**B**) Soluble protein content of the CR5 combination. (**C**) Soluble protein between CR4 and CR5 scions (up). (**D**) Soluble protein between CR4 and CR5 rootstock (down). Different uppercase letters indicate highly significant differences at the *p* < 0.01 level. Different lowercase letters indicate highly significant differences at the *p* < 0.05 level.

The temporal and spatial dynamics of soluble protein content in the two graft combinations were presented in Figure 2. In the CR4 combination (Figure 2A), the soluble protein content in the scion increased progressively over the 60-day period, reaching 84.13 mg/g at 60 days (*p* < 0.01), despite a transient decline to 61.80 mg/g at 40 days. The rootstock showed a more gradual but steady increase, culminating at 63.63 mg/g by 60 days (*p* < 0.05). For the CR5 combination (Figure 2B), the soluble protein content in the scion remained relatively stable (*p* > 0.05), fluctuating between 54.30 and 60.13 mg/g throughout the experiment. The graft rootstock protein content appeared largely unaffected, increasing steadily from 49.30 mg/g to 65.50 mg/g over 60 days (*p* < 0.01).

A comparative analysis of soluble protein content between the CR4 and CR5 combinations across different graft sites is detailed in Figure 2. Although no statistically significant difference was observed between the two scions’ soluble protein content in 60 days, the CR4 scion exhibited a significant increasing trend within 60 days after grafting from 53.13 mg/g to 84.13 mg/g. In contrast, CR5 scion soluble protein content kept relatively steady between 54.30 mg/g and 60.13 mg/g in 60 days. It could be inferred that the CR4 scion protein content variation was more sensitive than that of CR5 in the graft healing process, which may have increased scion compatibility (Figure 2C). The rootstocks of both combinations displayed a similar variation pattern (Figure 2D). The soluble protein content in both CR4 and CR5 rootstocks increased steadily from the time of grafting onward. No statistically significant difference in protein content was observed between the two rootstocks in 60 days.

**Figure 3:**
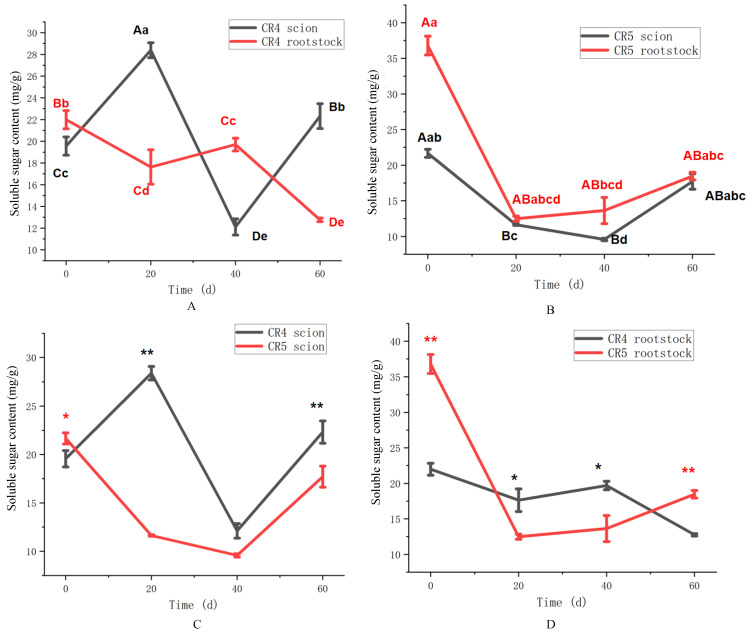
Soluble sugar content of CR4 and CR5 graft combinations. (**A**) Soluble sugar content of the CR4 graft combination. (**B**) Soluble sugar content of the CR5 graft combination. (**C**) Soluble sugar content between CR4 and CR5 scions. (**D**) Soluble sugar content between CR4 and CR5 rootstock. Different uppercase letters indicate highly significant differences at the *p* < 0.01 level. Different lowercase letters indicate highly significant differences at the *p* < 0.05 level. ** indicates a highly significant difference at the *p* < 0.01 level at the same time point. * indicates a highly significant difference at the *p* < 0.05 level at the same time point.

The spatiotemporal patterns of soluble sugar content for the two graft combinations are presented in Figure 3. In the CR4 combination (Figure 3A), the scion sugar content peaked early at 20 days (28.3 mg/g), declined to a minimum of 11.9 mg/g at 40 days, and subsequently recovered (*p* < 0.01). In contrast, the rootstock displayed a distinct and more stable decreasing trend over time (*p* < 0.01). The CR5 combination exhibited a significantly different soluble sugar profile (Figure 3B). The sugar content in the scion and rootstock both declined during the first 40 days (*p* < 0.05). By 60 days, the scion and rootstock showed a modest recovery to 17.7 mg/g and 18.3 mg/g, respectively. Overall, the temporal variation in soluble sugar was relatively synchronized in the CR5 combination, in contrast to the more divergent patterns seen in CR4.

A direct comparison of soluble sugar content between the CR4 and CR5 combinations across different graft sites was presented in Figure 3. The CR4 scion exhibited a significantly greater initial increase than did CR5 (11.6 mg/g) in soluble sugar, peaking at 28.6 mg/g by 20 days after grafting (*p* < 0.01) (Figure 3C). Subsequently, both scions showed a similar pattern (*p* > 0.05) of decline at 40 days, followed by partial recovery at 60 days. The CR4 scion maintained higher sugar levels than CR5 (22.4 vs. 17.6 mg/g) at 60 days (*p* < 0.01). The soluble sugar content in the rootstocks of both combinations decreased after grafting (Figure 3D). However, soluble sugar content decreased more significantly in CR5 rootstock than in that of CR4 at 20 days and 40 days after grafting (*p* < 0.05). By 60 days, the CR5 rootstock showed a slight increase, whereas the CR4 rootstock content did not exhibit a similar recovery (*p* < 0.01).

**Figure 4:**
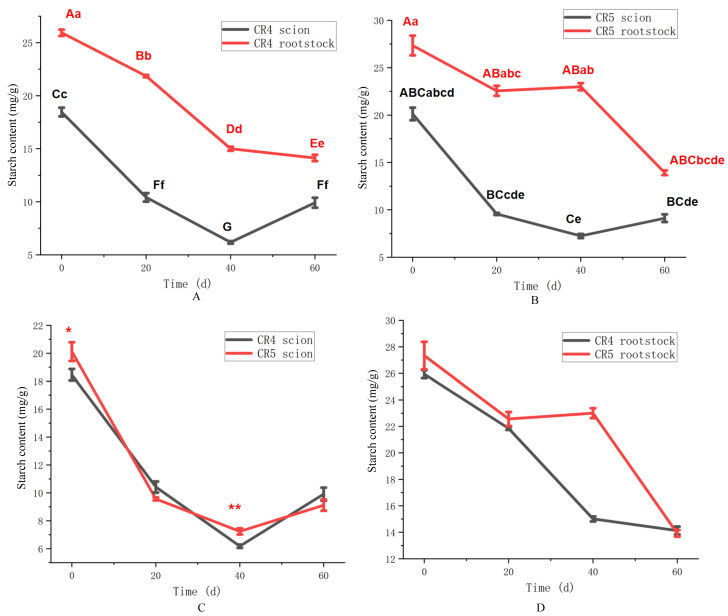
Starch content of CR4 and CR5 graft combinations. (**A**) Starch content of the CR4 graft combination. (**B**) Starch content of the CR5 graft combination. (**C**) Starch content between CR4 and CR5 scions. (**D**) Starch content between CR4 and CR5 rootstock. Different uppercase letters indicate highly significant differences at the *p* < 0.01 level. Different lowercase letters indicate highly significant differences at the *p* < 0.05 level. ** indicates a highly significant difference at the *p* < 0.01 level at the same time point. * indicates a highly significant difference at the *p* < 0.05 level at the same time point.

The temporal changes in starch content for the two graft combinations are shown in Figure 4. In the CR4 combination (Figure 4A), starch content in the scion and rootstock both decreased significantly following grafting (*p* < 0.01). Subsequently, the starch levels in the scion showed partial recovery by 60 days (*p* < 0.01), increasing to 9.8 mg/g from their lowest points at 40 days (6.1 mg/g). A similar overall declining trend was observed in the CR5 combination (Figure 4B). However, only the CR5 scion exhibited a limited recovery phase to 9.1 mg/g at 60 days from 7.1 mg/g at 40 days (*p* > 0.05). Notably, the starch content in the scions of both clones was lower than that in their respective rootstocks during the 60 days. And both scions and rootstocks continuously consumed starch for graft healing.

A direct comparison of starch content dynamics between the CR4 and CR5 combinations is presented in Figure 4. The scions of both clones exhibited highly similar temporal patterns in starch content (Figure 4C). Starting from initial levels of 18.5 mg/g (CR4) and 20.6 mg/g (CR5), starch declined sharply to minima of 6.2 mg/g and 7.2 mg/g, respectively, by 40 days after grafting. A slight recovery was observed in both by 60 days, reaching 9.9 mg/g (CR4) and 9.3 mg/g (CR5). According to Figure 4D, a continuous decline in rootstock starch content was observed over the 60-day period for both combinations. The depletion was notably more substantial in CR4, with its rootstock starch falling to 14.8 mg/g by 40 days and 22.8 mg/g in the CR5 rootstock. In summary, starch content was down-regulated across all tissues in both graft combinations during the healing process.

**Figure 5:**
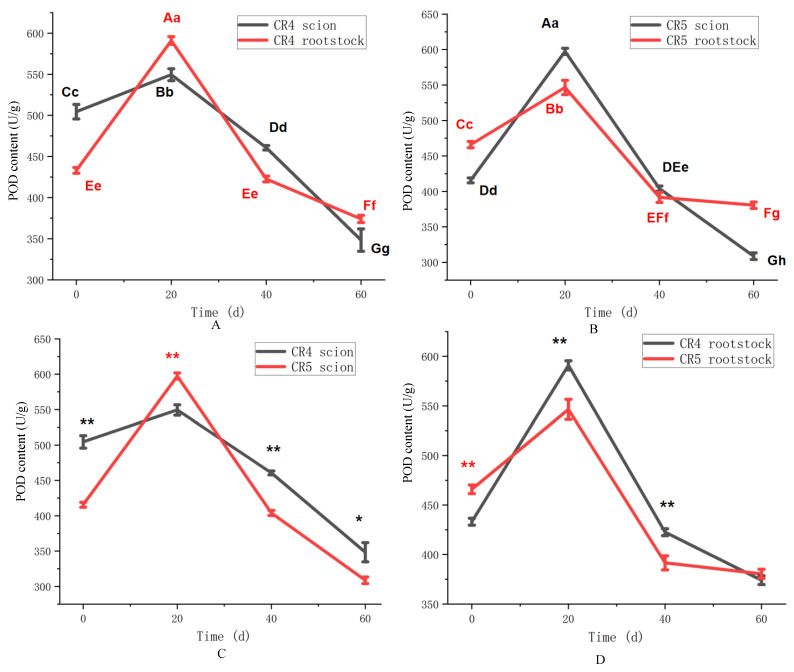
POD content of CR4 and CR5 graft combinations. (**A**) POD content of CR4 graft combination. (**B**) POD content of the CR5 graft combination. (**C**) POD content between CR4 and CR5 scions. (**D**) POD content between CR4 and CR5 rootstock. Different uppercase letters indicate highly significant differences at the *p* < 0.01 level. Different lowercase letters indicate highly significant differences at the *p* < 0.05 level. ** indicates a highly significant difference at the *p* < 0.01 level at the same time point. * indicates a highly significant difference at the *p* < 0.05 level at the same time point.

The activities of key regulatory enzymes—peroxidase (POD), polyphenol oxidase (PPO), and phenylalanine ammonia-lyase (PAL)—were measured and compared between the graft combinations. POD activity in all tissues of the CR4 combination (scion and rootstock) exhibited a similar pattern, characterized by an initial increase followed by a subsequent decline (Figure 5A). POD activity peaked at 20 days after grafting, reaching maximum values of 549.4 U/g in the scion and 590.9 U/g in the rootstock. Thereafter, POD activity decreased progressively, reaching minimum levels of 348.3 U/g and 374.1 U/g in the scion and rootstock, respectively, by 60 days. CR5 combination also exhibited a similarly varying pattern compared to CR4 (Figure 5B).

Although the temporal pattern of POD activity was similar between the CR4 and CR5 scions, the magnitude of change differed. The CR5 scion reached a significantly higher peak activity of 597.4 U/g at 20 days, compared to 549.5 U/g in CR4 (*p* < 0.01). Subsequently, the decline in POD activity was more pronounced in the CR5 scion, resulting in lower levels than CR4 at both 40 (*p* < 0.01) and 60 days (*p* < 0.05) (Figure 5C). The initial POD activity of CR4 scion at 0 days was also higher than that of CR5 at the *p* < 0.01 level. In the rootstocks, POD activity in both combinations increased by 20 days before declining. The peak activity was higher in the CR4 rootstock (590.9 U/g) than in its CR5 counterpart at 20 days (*p* < 0.01) (Figure 5D). Notably, the initial POD activity (0 day) in the CR4 scion (504.5 U/g) was substantially higher than that in the CR5 scion (415.6 U/g) (*p* < 0.01).

**Figure 6:**
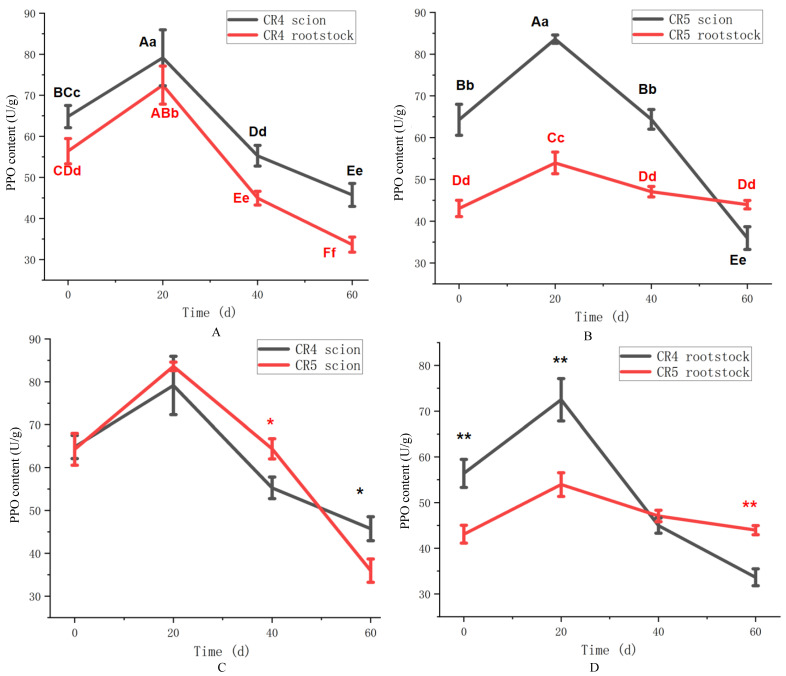
PPO content of CR4 and CR5 graft combinations. (**A**) PPO content of the CR4 graft combination. (**B**) PPO content of the CR5 graft combination. (**C**) PPO content between CR4 and CR5 scions. (**D**) PPO content between CR4 and CR5 rootstock. Different uppercase letters indicate highly significant differences at the *p* < 0.01 level. Different lowercase letters indicate highly significant differences at the *p* < 0.05 level. ** indicates a highly significant difference at the *p* < 0.01 level at the same time point. * indicates a highly significant difference at the *p* < 0.05 level at the same time point.

The activity of polyphenol oxidase (PPO) in the two graft combinations is shown in Figure 6, revealing distinct temporal patterns. CR4 PPO activity in the scion and rootstock increased initially, peaking around 20 days after grafting (*p* < 0.01), before declining over the remaining period (Figure 6A). Throughout the experiment, PPO activity was consistently highest in the scion, and the variation pattern was similar between scion and rootstock. While the CR5 scion also showed an initial increase and subsequent decline in PPO activity, the dynamics at the rootstock diverged markedly (Figure 6B). The CR5 rootstock PPO activity increased slightly over 20 days, and was significantly lower than that in the CR5 sicon (*p* < 0.01). This asynchronous variation between the scion and the rootstock suggests a weaker physiological connection in the CR5 combination compared to CR4.

A direct comparison of PPO activity between the CR4 and CR5 combinations across different graft components is detailed in Figure 6. Both scions exhibited a similar temporal pattern of PPO activity. However, the activity in the CR5 scion was higher than in CR4 at 40 days, but became lower by 60 days (*p* < 0.05) (Figure 6C). Rootstock PPO activity in both combinations followed an increase-then-decline pattern (Figure 6D). The CR4 rootstock showed higher activity at 20 days (72.4 vs. 53.9 U/g in CR5) (*p* < 0.01), whereas the CR5 rootstock exhibited higher activity by the endpoint at 60 days (43.9 vs. 33.6 U/g in CR4) (*p* < 0.01). According to the PPO variation pattern, it could be inferred that 20 days after grafting was the key period of graft healing.

**Figure 7:**
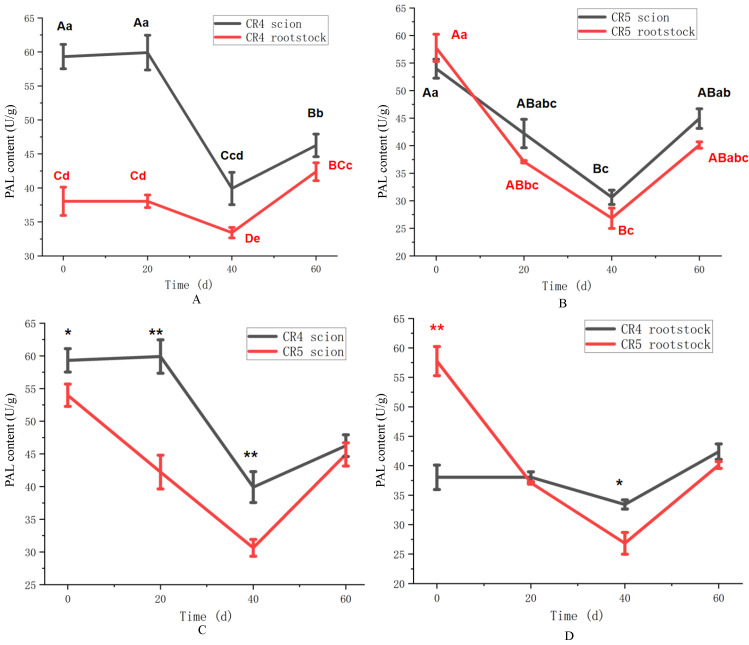
PAL content of CR4 and CR5 graft combinations. (**A**) PAL content of the CR4 graft combination. (**B**) PAL content of the CR5 graft combination. (**C**) PAL content between CR4 and CR5 scions. (**D**) PAL content between CR4 and CR5 rootstock. Different uppercase letters indicate highly significant differences at the *p* < 0.01 level. Different lowercase letters indicate highly significant differences at the *p* < 0.05 level. ** indicates a highly significant difference at the *p* < 0.01 level at the same time point. * indicates a highly significant difference at the *p* < 0.05 level at the same time point.

The activity of phenylalanine ammonia-lyase (PAL) exhibited distinct spatial and temporal patterns in the two graft combinations, as shown in Figure 7. In the CR4 combination (Figure 7A), PAL activity in the scion remained at a high level (59.9 U/g) until 20 days, decreased to a minimum of 39.9 U/g at 40 days (*p* < 0.01), and then partially recovered to 46.3 U/g by 60 days (*p* < 0.01). In contrast, the rootstock PAL activity declined continuously over the first 40 days, reaching 33.1 U/g, and showed only marginal recovery thereafter (41.8 U/g at 60 days). The rootstock PAL activity followed a trend similar to that of the scion, albeit at consistently lower absolute levels throughout the 60-day period. In the CR5 combination (Figure 7B), a different pattern was observed. PAL activity in the scion and rootstock all decreased from their initial maxima at 0 days (53.9 and 57.7 U/g, respectively) to distinct minima at 40 days (*p* < 0.01) (30.6 and 26.8 U/g, respectively), followed by a moderate increase by 60 days.

A comparative analysis of PAL activity between the CR4 and CR5 combinations across different graft sites is presented in Figure 7. The CR4 scion maintained significantly higher PAL activity than the CR5 scion throughout the initial 40 days (Figure 7C). This indicates a more sustained and active phenylpropanoid metabolism in the CR4 scion during graft healing, especially at 20 days and 40 days (*p* < 0.01). At the rootstock, PAL activity in both combinations declined from initial levels (Figure 7D). While the CR5 rootstock initially showed higher activity than CR4 at 0 days, it subsequently decreased sharply. In contrast, the decline in the CR4 rootstock was more gradual. By 60 days, the CR5 union activity showed a modest increase, but overall, the CR4 rootstock exhibited a more stable activity profile over time.

### 3.4. Differential Accumulation of Phenylpropanoids

**Figure 8:**
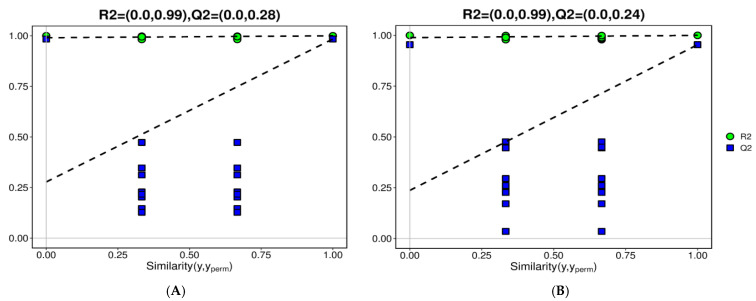
OPLS-DA permutation test plot of the metabolome analysis. (**A**) CR5up60-vs-CR4up60.POS. (**B**) CR5up60-vs-CR4up60.NEG.

To investigate the relationship between graft compatibility and secondary metabolism, the relative abundances of phenylpropanoids and flavonoids in the scions were profiled at 60 days after grafting. In the metabolome analysis, both positive ion mode (POS) and negative ion mode (NEG) ionization methods were used, and the data from both modes were analyzed separately. According to the cross-validation parameters of the OPLS-DA (orthogonal partial least squares–discriminant analysis) model, the Q2 values of both POS and NEG were higher than 0.9, which represent a reliable result (Table 3). In the OPLS-DA permutation test plot of POS and NEG. All Q2 values after permutation were consistently lower than the original Q2 values, indicating a reliable result (Figure 8). The metabolome analysis screened 601 differentially accumulated metabolites, including 351 up-regulated metabolites and 250 down-regulated metabolites in the CR4 scion.

Phenylpropanoids exhibiting both high ion abundance and significant differential accumulation between the two graft combinations were identified (Table 4). FDR values of most selected metabolites were lower than 0.1, which decreased the risk of false positives. According to the comparative analysis, five flavonoids were significantly more abundant in CR4 compared to CR5: dihydromyricetin, kaempferol, astragalin, quercetin 3-glucoside and quercetin (log_2_FC value = 1.07, 2.32, 2.44, 2.14 and 2.10). In contrast, only two flavonoids, kaempferide and prunetin, accumulated to higher levels in the CR5 scion (log_2_FC = −1.89 and −3.72, respectively). Beyond flavonoids, the CR4 scion also demonstrated greater accumulation of specific phenylpropanoid pathway derivatives, including the lignan podophyllotoxin, as well as coniferyl aldehyde and coniferyl alcohol (log_2_FC value = 1.11, 2.56 and 1.90).

## 4. Discussion

Grafting compatibility is a primary determinant of graft success. Research on litchi, pecan, *Camellia oleifera*, and *C. paliurus* all considered that graft combinations can significantly enhance the efficiency of clonal cultivation [8,9,13,14,15,16,17,18]. Although survival rate and growth of grafted plants were both critical indicators for evaluating grafting compatibility, graft survival rate represented the core assessment parameter [19]. The scion mainly regulated vascular bundle formation at the rootstock–scion junction [9], while the rootstock exerted a more pronounced influence on the growth of grafted plants [20,21]. Previous research on *C. paliurus* reported an average grafting survival rate of approximately 53.49% [7]; this study revealed a marked divergence between two clonal scions. By 60 days after grafting, the CR4 combination achieved a 60% survival rate, significantly higher than the 35% observed for CR5. This agreed with our previous research that scion combination significantly regulated graft survival rate [8]. Given that both combinations employed the same rootstock, the observed disparity in survival rate is likely attributable to intrinsic physiological differences between the CR4 and CR5 scion clones. The difference was inferred to be associated with different genetic traits.

The graft healing process is known to demand substantial carbohydrate resources [22,23]. Our results showed that starch content in both CR4 and CR5 combinations decreased significantly during the initial 40 days and reached minimal levels at 40 days. This early decline reflects the high metabolic cost of graft union healing. The first 20 days after grafting are considered to be the critical period of graft union formation and sufficient nutrient supply [16]. Soluble sugar content of the CR4 combination peaked at 20 days, obtaining greater nutrient availability than CR5 at the key healing stage. As healing progressed past 40 days, the onset of vascular reconnection likely restored resource allocation balance, leading to a rebound in soluble sugar and starch content. Li et al. (2021) [7] also reported that sugar and starch contents of *C. paliurus* rootstocks and scions first decreased within 40 days and then increased over time. It could be inferred that the critical healing period for grafting is the first 40 days.

Soluble proteins play vital roles in plant wound healing, with their concentration often indicating the metabolic vigor of plant tissues [16,24,25,26]. In the *C. paliurus* graft combination, soluble protein levels in the scion exhibited an “increase–decrease–recovery” pattern, peaking at 20 days before declining to a minimum at 40 days, and then rising again. Li et al. (2021) [7] also found that soluble protein content first increased within 40 days and then decreased by 70 days of *C. paliurus* scions. And the research on pecan grafting agreed that soluble protein content increased more within the first 20 days in the compatible graft combination for graft healing [16]. In this research, although the difference was not statistically significant, the more compatible CR4 scion maintained higher soluble protein content than CR5, meaning stronger healing ability.

Peroxidase (POD) plays a multifaceted role in graft healing, functioning both in scavenging free radicals to mitigate membrane lipid peroxidation and in promoting lignin biosynthesis [27,28]. Enhanced POD activity has been associated with improved graft union formation, survival, and subsequent growth [29,30]. The research on *Camellia oleifera* has found that POD content significantly accumulated in the compatible graft combination 20 days after grafting [18]. Meanwhile, the research on *Prunus cerasifera* indicated that peroxidase serves as the early indicator of physiological compatibility [31]. In this study, POD activity in both CR4 and CR5 combinations followed this expected pattern, peaking at 20 days before decreasing. The initial surge is likely a defensive response to oxidative stress at the wounded interface. The CR5 combination exhibited a transiently higher POD peak at 20 days, and the CR4 combination maintained significantly higher activity during most other periods. This sustained elevation in POD activity in CR4 may suggest more robust and prolonged antioxidant capacity and healing progression, correlating well with its higher final survival rate.

Polyphenol oxidase (PPO), a key enzyme in phenylpropanoid metabolism, contributes to graft healing through multiple mechanisms. Early in the process, PPO oxidizes phenols at the graft interface to quinones, which polymerize to form a protective barrier [32]. Later, PPO facilitates lignin synthesis and vascular tissue differentiation [33]. A characteristic rise and fall in PPO activity is generally observed during successful healing, whereas persistently high activity is often linked to incompatibility [34]. In our experiment, scion and rootstock PPO activity in both combinations increased and then decreased, likely reflecting an early stress response to graft-induced reactive oxygen species. However, a critical divergence was observed in the rootstock of the two combinations. While CR4 rootstock PPO activity followed the typical unimodal trend, the CR5 rootstock PPO activity was higher than that of the CR4 scion in 60 days, indicating incompatibility of the graft union to some extent.

PAL occupies a central position in the phenylpropanoid pathway [35]. It catalyzes the deamination of phenylalanine to cinnamic acid, a pivotal precursor for monolignols such as coniferyl, sinapyl, and p-coumaryl alcohols. These monolignols are subsequently oxidized (e.g., by POD) and polymerized to form lignin, a process critical for vascular tissue development and graft union strength [36]. Consequently, PAL activity is often positively correlated with graft survival [37], while previous studies also demonstrated that PAL is associated with plant graft incompatibility [31]. This is because PAL acts as the key enzyme of phenolic compounds biosynthesis, which might lead to graft incompatibility [35]. In this study, PAL activity in both graft combinations displayed a “decrease–recovery” pattern, reaching a nadir at 40 days. The initial decline may correspond to the early phase of callus formation, and the subsequent rise after 40 days likely marks the onset of active lignification, reinforcing the nascent graft union—a dynamic consistent with observations in other grafted species [38]. Despite differences in PAL activity between CR4 and CR5 becoming less pronounced by 60 days, the CR4 combination exhibited significantly higher activity during the critical early and middle stages (0–40 days), particularly in the scion. This suggests that CR4 possesses a more robust and rapidly activated phenylpropanoid metabolism, capable of supplying lignin precursors earlier and in greater abundance to facilitate union healing. The inhibitory effect of phenolic acids on graft healing appears to be a secondary factor. Approximately 20 days after grafting is considered the critical period, and a deeper survival analysis of this period should be performed in further research.

Phenylpropanoids have emerged as potential biochemical markers for graft compatibility [8,39]. Prior studies on *Vitis* have linked intermediates of lignin biosynthesis, such as ferulic acids and caffeic acids, to compatibility [40,41]. And secondary metabolites, including phenylpropanoids, were found to accumulate in compatible litchi graft combinations [15]. Similarly, our findings align with this, showing significantly higher levels of the lignin precursors coniferyl aldehyde and coniferyl alcohol, as well as the lignan podophyllotoxin, in the more compatible CR4 scion. This accumulation points to enhanced flux through the phenylpropanoid pathway in CR4, which likely accelerates lignin deposition and graft union maturation. Higher levels of the lignin precursors and the lignan were consistent with higher PAL activity in CR4 scions.

Flavonoids, another major class of phenylpropanoid derivatives, appear to play a dual role in graft healing. According to research on walnut and *Vitis*, flavonoids and phenolic acids contribute to antioxidant defense but, at high concentrations, may potentially interfere with vascular differentiation [40,41,42]. In our study, the metabolic profile was more complex: dihydromyricetin and quercetin derivatives accumulated to higher levels in the CR4 scion, kaempferide and prunetin were more abundant in CR5. The overall up-regulation of flavonoid biosynthesis in the compatible CR4 combination, positioned downstream of the PAL-catalyzed step, indicates a broadly enhanced phenylpropanoid metabolism. When integrated with the observed patterns of PAL and POD activity, these metabolite data strongly suggest that graft compatibility in *C. paliurus* is closely associated with the capacity of the scion to sustain an active and coordinated phenylpropanoid metabolism and, crucially, structural reinforcement of the graft union.

Although phenylpropanoid metabolism has been identified as regulating *C. paliurus* graft compatibility by biochemical and metabolomic analysis in this research. Additional grafting combination trials of *Cyclocarya paliurus* are needed to further confirm the research findings. The effects of plant hormones on *C. paliurus* graft compatibility warrant further investigation [43,44,45]. Identification of key transcription factors and other differentially expressed genes [46,47,48,49,50,51] is also needed to further uncover the mechanism of *C. paliurus* graft incompatibility.

## 5. Conclusions

This study successfully identified a *Cyclocarya paliurus* scion clone with superior graft compatibility, exhibiting a final survival rate 25% higher than the control. The enhanced compatibility of CR4 was associated with distinct physiological and biochemical profiles during graft healing. Specifically, the CR4 combination maintained consistently higher levels of soluble proteins and key enzyme activities (POD and PAL). Furthermore, metabolic profiling revealed greater accumulation of phenylpropanoid pathway products—including specific lignans, lignin precursors (coniferyl aldehyde and alcohol), and several flavonoid compounds—in the CR4 scion. The findings indicate that the superior graft scion in *C. paliurus* is closely linked to a more active and sustained phenylpropanoid metabolism. This metabolic capacity likely facilitates more efficient wound healing, lignin deposition for vascular reconnection, and stress mitigation at the graft union. This work establishes a valuable foundation for advancing clonal breeding and efficient grafting propagation of *C. paliurus*. Additional grafting combination trials and transcriptome analysis are still needed to uncover the mechanism of *C. paliurus* graft incompatibility further.

## Figures and Tables

**Table 1:** Descriptive statistics for scion physiological and biochemical indicators.

Indicators	Day	N	CR4		CR5	
			Mean Value	Coefficient of Variation	Mean Value	Coefficient of Variation
Soluble sugar (mg/g)	0	9	22.09 ± 0.85	0.12	25.4 ± 2.91	0.34
	20	9	23.03 ± 1.6	0.21	12.78 ± 0.39	0.09
	40	9	13.7 ± 1.56	0.34	11.83 ± 0.7	0.18
	60	9	16.97 ± 1.42	0.25	16.22 ± 0.96	0.18
Starch (mg/g)	0	9	21.45 ± 1.15	0.16	23.82 ± 1.1	0.14
	20	9	14.64 ± 1.82	0.37	17.49 ± 2.01	0.34
	40	9	10.81 ± 1.28	0.36	14.38 ± 2.31	0.48
	60	9	12.12 ± 0.62	0.15	11.66 ± 0.7	0.18
Soluble protein (mg/g)	0	9	42.39 ± 2.99	0.21	53.54 ± 2.47	0.14
	20	9	59.43 ± 2.07	0.10	52.02 ± 2.51	0.14
	40	9	49.95 ± 4.93	0.30	46.58 ± 5.01	0.32
	60	9	70.97 ± 3.34	0.14	57.56 ± 2.37	0.12
PPO (U/g)	0	9	58.98 ± 1.68	0.09	48.25 ± 4.17	0.26
	20	9	75.81 ± 1.73	0.07	57.65 ± 7.04	0.37
	40	9	49.03 ± 1.67	0.10	48.06 ± 4.6	0.29
	60	9	39.29 ± 1.85	0.14	41.74 ± 1.6	0.11
POD (U/g)	0	9	496.67 ± 17.54	0.11	444.26 ± 7.56	0.05
	20	9	567.21 ± 6.35	0.03	576.37 ± 7.89	0.04
	40	9	439.58 ± 5.66	0.04	399.64 ± 2.52	0.02
	60	9	384.05 ± 12.25	0.10	357.62 ± 12.35	0.10
PAL (U/g)	0	9	45.09 ± 3.64	0.24	55.57 ± 0.76	0.04
	20	9	44.05 ± 4.04	0.28	39.34 ± 0.88	0.07
	40	9	34.38 ± 1.62	0.14	26.09 ± 1.51	0.17
	60	9	39.62 ± 2.44	0.19	36.3 ± 3.25	0.27

**Table 2:** ANOVA of variance between CR4 and CR5 graft combinations.

Sig.	PPO	POD	PAL	Soluble Sugar	Starch	Soluble Protein
Scion						
Homogeneity of Variance	0.129	0.7	0.87	0.1	0.23	<0.05
Days × Clone	<0.01	<0.01	<0.01	<0.01	<0.01	-
Rootstock						
Homogeneity of Variance	0.09	0.19	0.09	0.12	<0.05	<0.05
Days × Clone	<0.01	<0.01	<0.01	<0.01	-	-
CR4						
Homogeneity of Variance	0.048	0.07	0.25	0.15	0.19	<0.05
Days × Site	0.53	<0.01	<0.01	<0.01	<0.01	-
CR5						
Homogeneity of Variance	0.37	0.24	<0.05	<0.05	<0.05	<0.05
Days × Site	<0.01	<0.01	-	-	-	-

**Table 3:** Cross-validation parameters of the OPLS-DA model.

Type	Pair	R2X (cum)	R2Y (cum)	Q2 (cum)
OPLS-DA	CR5up60-vs-CR4up60.POS	0.732	1	0.984
	CR5up60-vs-CR4up60.NEG	0.628	1	0.954

**Table 4:** Phenylpropanoids and flavonoids between CR5 and CR4 graft scions of *Cyclocarya paliurus* (Batal.) Iljinsk in 60 days.

	Individuals	CR5 (Ion Abundance)	CR4 (Ion Abundance)	log2 FC	*p* Value	VIP	FDR
Flavonoids	Dihydromyricetin	12,004,463.56 ± 906,356.17 b	25,233,779.97 ± 3,440,742.61 a	1.07	0.02	1.63	0.07
	Kaempferol	11,162,376.25 ± 755,078.49 b	55,574,300.58 ± 1,841,952.99 a	2.32	<0.001	3.20	0.02
	Kaempferide	22,284,592.09 ± 601,141.52 a	5,995,953.37 ± 786,828.53 b	−1.89	<0.001	1.93	<0.01
	Astragalin	7,281,585.79 ± 361,422.86 b	39,412,345.34 ± 717,245.32 a	2.44	<0.001	2.72	<0.01
	Quercetin 3-glucoside	6,943,479.61 ± 582,818.44 b	30,662,364.89 ± 7,581,074.54 a	2.14	0.04	2.15	0.09
	Quercetin	29,280,458.74 ± 1,298,925.23	125,479,860.88 ± 3,858,199.1 a	2.10	<0.001	4.71	<0.01
	Prunetin	16,086,648.89 ± 4,900,271.75 a	1,220,468.49 ± 5400.47 b	−3.72	0.04	1.69	0.11
Lignans	Podophyllotoxin	88,149,670.02 ± 1,379,653.33 b	190,577,068.35 ± 1,766,034.38 a	1.11	<0.001	4.86	<0.01
Coniferyl alcohol derivatives	Coniferyl aldehyde	3,023,932.34 ± 508,035.87 b	17,820,064.11 ± 4,351,054.22 a	2.56	0.03	1.72	0.08
	Coniferyl alcohol	44,193,907.08 ± 1,918,441.04 b	165,481,568.54 ± 7,575,573.63 a	1.90	<0.001	5.27	<0.01

Lowercase letters indicate a significant difference at the 0.05 level.

## Data Availability

The data presented in this study are available on request from the corresponding author, and the data are not publicly available due to privacy restrictions.

## Supplementary Materials

The following supporting information can be downloaded at: https://www.mdpi.com/article/10.3390/plants15101536/s1. Figure S1. Morphological changes in scions during the graft healing process of Cyclocarya paliurus seedlings ((A) CR4. (B) CR5).
