# Supplementary material for: Comparative Analysis of Physiological and Biochemical Responses Between Compatible and Incompatible Graft Combinations of Cyclocarya paliurus
Source: Plants (Basel). 2026 May 18;15(10):1536. doi: 10.3390/plants15101536 (PMC13210612; doi:10.3390/plants15101536)
Supplement: Supplementary file 1 [file plants-15-01536-s001.zip › plants-4252375-supplementary.pdf]

supplementry materials

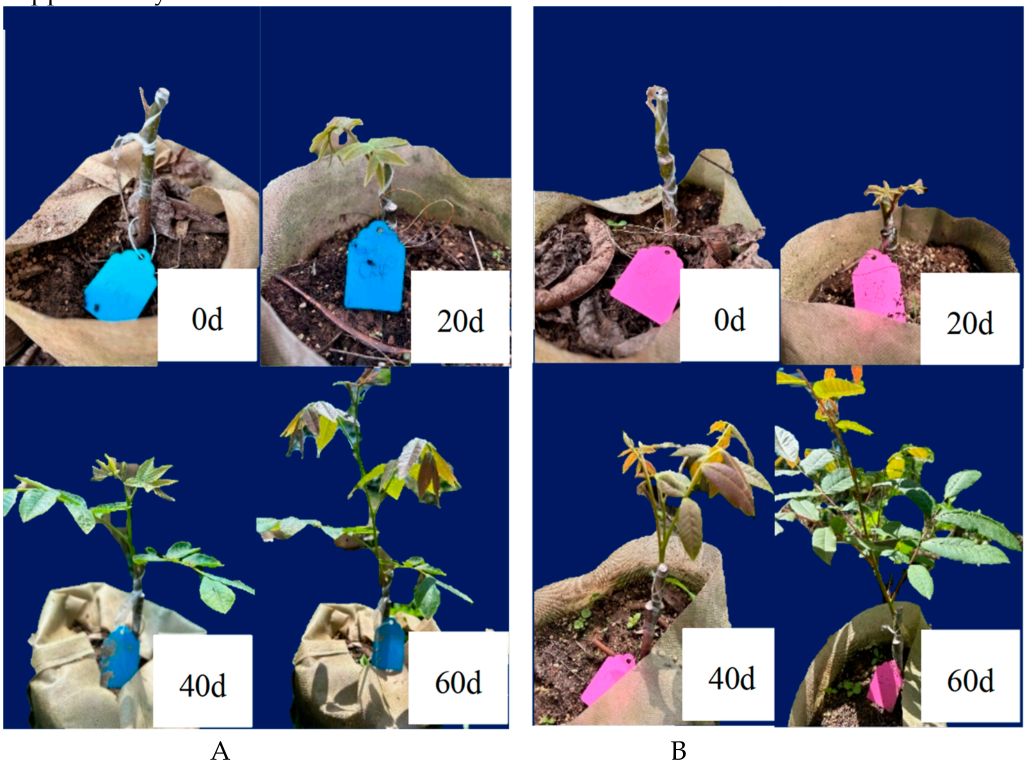

Figure S1. Morphological changes in scions during the graft healing process of Cyclocarya paliurus seedlings ((A) CR4. (B) CR5)
